# Land Use Changes and Cluster Identification of Dengue Hemorrhagic Fever Cases in Bandung, Indonesia

**DOI:** 10.3390/tropicalmed5020070

**Published:** 2020-05-02

**Authors:** Sri Yusnita Irda Sari, Yessika Adelwin, Fedri Ruluwedrata Rinawan

**Affiliations:** Department of Public Health, Faculty of Medicine, Universitas Padjadjaran, Bandung 40161, Indonesia; yessika.adelwin@yahoo.com (Y.A.); f.rinawan@unpad.ac.id (F.R.R.)

**Keywords:** dengue hemorrhagic fever, normalized difference vegetation index, SaTScan, spatiotemporal analysis

## Abstract

Dengue Hemorrhagic Fever (DHF) in Indonesia has increased steadily with Bandung as a hyper-endemic area holding a high number of cases for years. This study aimed to identify cluster areas and their correlation with land use changes which was indicated by changes of Normalized Difference Vegetation Index (NDVI). Hospital surveillance of 28,327 cases during 2008–2013 was geo-coded into sub-district levels and analyzed to find cluster areas over time and space using SaTScan and ArcGIS. Spearman correlation was used to analyze NDVI with Incidence Rate (IR) in each area. IR of DHF cases tended to increase over 6 years during high precipitation period. Cases were concentrated in several cluster areas in 2009 then moved to eastern part of the city in 2013. NDVI had negative correlation with IR in 2008 (r = −0.258; p = 0.001) and positive correlation in 2012 (r = 0.193; p = 0.017). Clear geographical pattern by cluster identification overtime is beneficial for targeting appropriate vector-control program.

## 1. Introduction

Dengue remains the most prevalent and rapidly spreading mosquito-borne viral infection among infectious diseases. About half of the world’s population is at risk to acquire this infection, particularly in tropical countries. The transmission during the past 50 years is related to some eco-bio-social determinants such as unprecedented population growth, increasing movement of people and the viruses, uncontrolled urbanization, land use and climate change as well as breakdown in public health infrastructure and vector control programs [1]. As one of dengue endemic countries in Asia, Indonesia reported an average number of 129,435 dengue cases during 2004 to 2010. This number was the second highest after Brazil [2]. Annual incidence rate increased from 0.05/100,000 in 1968 to 37/100,000 in 2013. However, Case Fatality Rate (CFR) declined from 41% in 1968 to 0.73% in 2013. The incidence of Dengue Hemorrhagic Fever (DHF) as the more severe form of dengue infection, increased among the age group of >15 years old since 1999 [3].

West Java Province, as the most populous area in Indonesia, has been reporting the highest number of dengue cases in Indonesia for years. In 2012, there were 19,663 dengue cases reported, with CFR of 0.85%. Most cases were found in Bandung, the capital city of West Java province which has the second highest population density rate (14,847 people/km^2^) after Jakarta, the capital city of Indonesia (15,341 people/km^2^). After the last dengue outbreak in 2009 with 6678 cases reported being hospitalized for DHF, the number of cases had been found to steadily increase during the period of 2012–2013 [4].

A cohort study conducted among adult volunteers from two textile factories in Bandung showed that the incidence of symptomatic DHF was 18 cases per 1000 person–years. The investigators also estimated infection rate of 56 cases per 1000 person-years for asymptomatic/mild infection cases [5]. DENV-2 virus was identified as the pre-dominant serotype. In secondary infection DENV-3 was found to be more dominant (47.1%) followed by DENV-1 (40.9%), DENV-2 (30%) and DENV-4 (27%) [6].

The pattern of dengue outbreak in hyper-endemic area such as Bandung remains unpredictable. The abundance of susceptible host, mosquito as the vector, identified 4 serotypes of dengue virus or the increase of clustered or scattered area including plausible determinants such as demography and environmental aspects play an important role to determine high risk and low risk areas. Vegetation is one of environmental factors in which many water receptacles, e.g., tree axils, decaying leaves or paddy field are very potential as habitats for the mosquitoes in their aquatic phase [7,8]. Land use changes due to rapid and uncontrolled urbanization may contribute to change the pattern of DHF cases. Normalized Difference Vegetation Index (NDVI) as an index which calculates the greenness of one area can be used to analyze the change of land use trend overtime.

Recently, spatiotemporal or space-time analysis is commonly used to evaluate clustering of various diseases. Syndromic surveillance or monitoring the frequency of illnesses with a specific set of clinical features is an investigational approach with which public health office will be able to monitor disease indicators in real time or near real time, in order to detect outbreaks of diseases earlier. This analysis also improves our capability to detect changes of the disease pattern at a fine geographical area. Monitoring of trends through a routine surveillance of dengue cases will support the public health office to conduct public health responses which cover not only disease prevention such as effective vector control management, but also control measures such as setting up early detection systems to minimize morbidity and mortality [8]. This study aimed to investigate spatiotemporal pattern to identify cluster area of DHF cases and the correlation of NDVI as environmental risk factor of land use changes in Bandung.

## 2. Materials and Methods

### 2.1. Study Area

Bandung is located at 107°36″ East longitudes and 6°55′ South latitudes, with an average height of ±791 meter above sea level. Bandung’s highest point is in the northern part which is topographically mountainous (±1050 m), whereas the lowest point is ±675 m in the southern part. The climate is mostly influenced by the region’s mountainous topography and this causes the weather to become humid and cool. As a tropical country in the equator line, Indonesia typically enters dry season in May–October and wet season in November–April with an average temperature of 23 °C (18.8–29.6 °C). However, the period of dry and wet season have changed due to the effect of global climate change and this causes the temperature to reach above 30 °C. Bandung is divided into 30 districts and 151 sub-districts which serve as the smallest unit of governmental structure with a population of about 2,470,802 in 2014. The region covers 16,731 ha which are mostly residential areas (83.4%). With only 8.3% wetlands/ponds and 4.9% farm/garden, the land use change is rapidly observed due to urbanization and city development plan.

### 2.2. Data Collection

**Figure 1:**
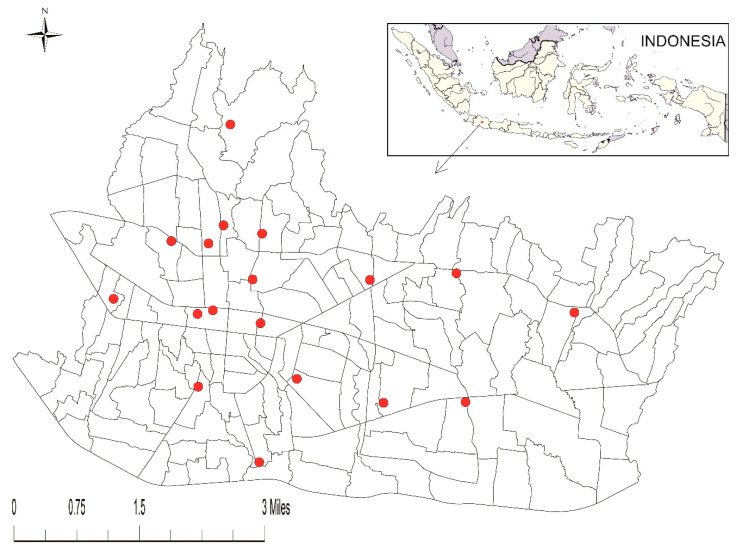
Study site and the location of the hospitals.

The data of Dengue Hemorrhagic Fever (DHF) and Dengue Shock Syndrome (DSS) were compiled in the period of 2003–2013 from Disease Prevention Unit and Environmental Health of Bandung Health Office. Due to the difference in the surveillance system used in that period, the data retrieved in 2003–2008 were only available as monthly cumulative number of reported DHF. Passive dengue surveillance system using daily data were established in 2008. Therefore, the analysis of spatiotemporal cases was calculated between the period of 2008–2013. Severe dengue cases were regularly reported by 18 hospitals to health office using a monthly report form. The form consists of information such as: name, sex, age, address, name of parents (for children), type of diagnosis (DHF, DSS), date of diagnosis, platelet count, hematocrit count, IgG, IgM and outcome (alive or dead). However, not every hospital was able to perform ELISA test. Therefore, neutralized antibody like IgG and IgM examination was not always conducted since it is labor-intensive and costly. The location of 18 hospitals in the study site was illustrated in Figure 1.

NDVI was documented from Landsat 7 Enhanced Thematic Mapper plus (ETM+) LIT, Scan Line Corrector (SLC)-off images dated 18 July 2008, 24 January 2008, 30 August 2012 and 27 June 2012 with less than 10% cloud cover from USGS website. NDVI was calculated using reflectance value with the following formula: (band 4 − band 3)/(band 4 + band 3) in bandmath. NDVI result images were classified by range to define the density of −1 to 0 (no vegetation); 0 to 0.25 (low density); 0.25 to 0.5 (medium density); 0.5 to 0.75 (high density) and 0.75 to 1 (very high density). Furthermore, density slice divided the range by color. These processes were done using ENVI 4.8.

### 2.3. Data Analysis

The data were analyzed from aggregated number of DHF cases from each sub-district. A retrospective space-time permutation scan statistic with discrete Poisson probability model was used to detect possible clusters (SaTScan software package v9.3, www.satscan.org). The center of window was the centroid of each sub-district and latitude/longitude from all geometric centers was obtained using ArcGIS geocoding (ArcGIS version 10.2, ESRI, Inc., Redlands, CA, USA). Circular window scanned the areas with high or low rates, with time aggregation units of 1 day. Maximum Spatial Cluster Size was set to 10% of population at risk, with maximum Temporal Cluster Size of 10% of study period. Reported Clusters were defined only for clusters smaller than 10% of the population at risk, in order to get better resolution. Monte Carlo replication was set at 999 permutations and statistical significance at 0.05. The Ethical clearance was approved by the Ethical committee Faculty of Medicine, Universitas Padjadjaran, Bandung, Indonesia.

## 3. Results

**Figure 2:**
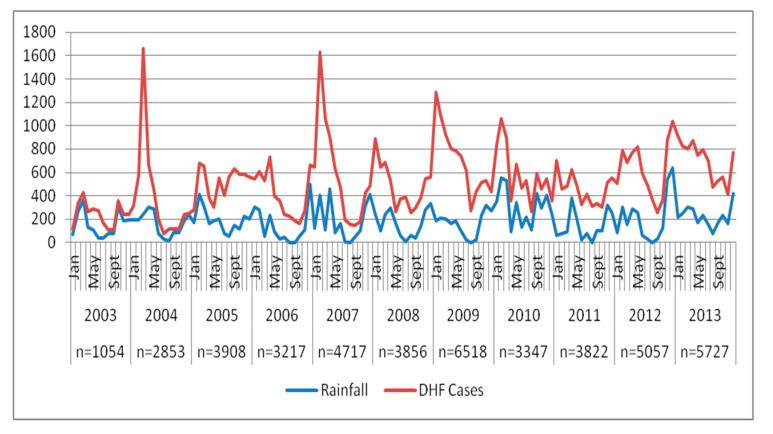
Number of reported DHF cases in Bandung from 2003 to 2013.

In Bandung, during the period of 2008–2013, a total of 28,327 hospitalized DHF and DSS cases were reported. Between 2003 and 2008, an outbreak was reported only every 2–3 years. However, after 2009 the number of DHF cases steadily increased, reaching its highest peak in January–March in concordance with the peak of wet season and the number decreased in August- November during dry season, as shown in Figure 2. The number of cases was not different between male and female group. Most cases were reported among group of elementary school age to late teenagers (5–19 years old) and adult (20–59 years old), with a percentage of 42.3% and 38.1%, respectively. These age groups spend their time mostly in schools or workplaces during daytime. Children under five and elderly people who spend most of their time at home comprised only 19.6% of the cases. DSS cases were reported only 1.5% of total cases and were found to slightly increase from 0.8% in 2008 to 2.5% in 2013. An outcome of death was only reported among 0.1% of cases. However, most of the outcomes were unreported (46.9%). The diagnosis of DSS was highly associated with risk of death (OR = 57.16; CI = 30.39–107.48; *p* = 0.001).

**Figure 3:**
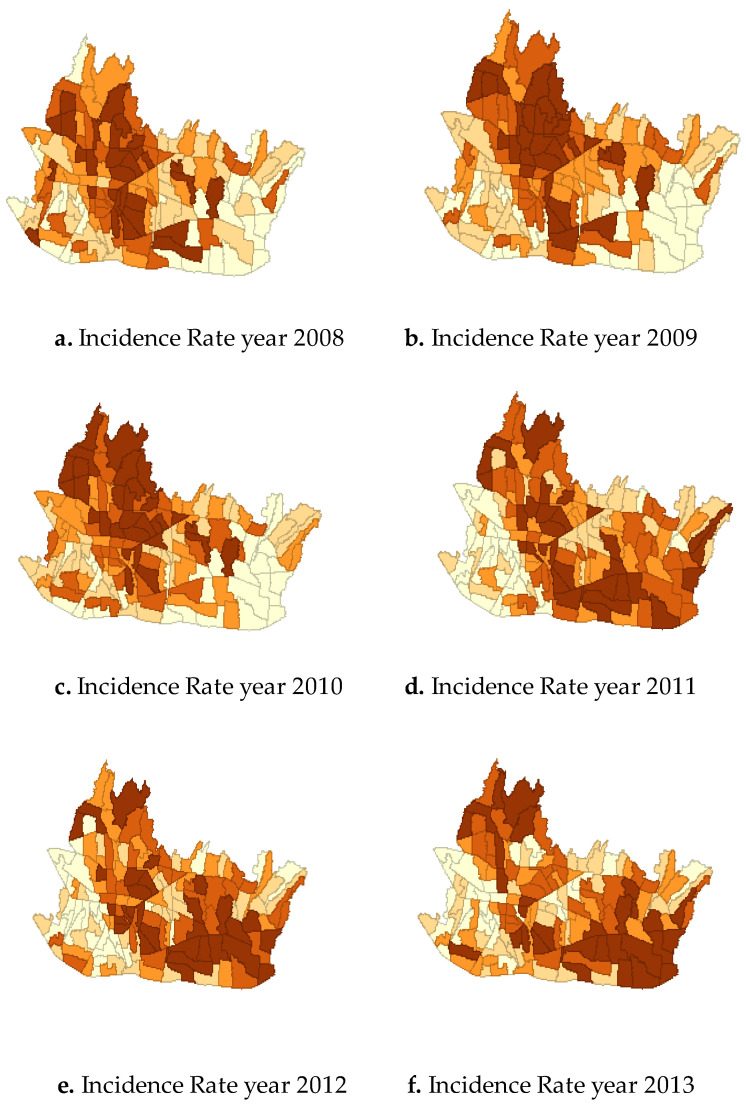
Variation of DHF Incidence Rate in Bandung from 2008 to 2013.

Incidence Rate (IR) of DHF cases per 1000 inhabitants had different pattern from 2008 to 2013 which is described in Figure 3. The numbers of cases were plotted in each area using quintile map which is divided into 5 quintiles. The map showed that IR in each area gradually changed with time. In 2008 and 2010, high number of IR was found predominantly in the northern part of the city. Interestingly, from 2011 the cases spread to the eastern and southern parts of the city. Eventually, in 2012 and 2013 high IR was found mostly in southeast area, while certain areas in the northern part persistently showed high IR from 2008 to 2013.

**Figure 4:**
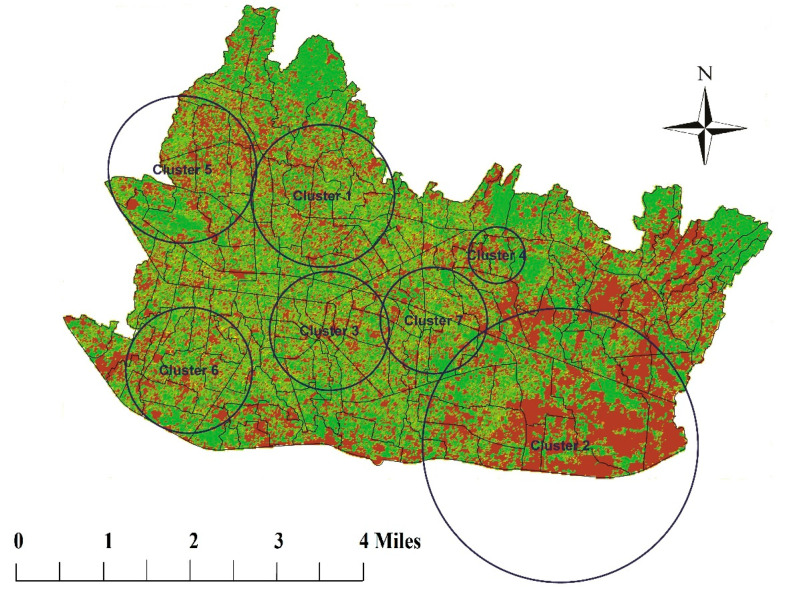
Cluster area of DHF cases and differences of NDVI between 2008 and 2012.

Land use changes in Bandung are mostly caused by urbanization, when more residential/housing areas were extended from the city center to the eastern and southern parts of the city. Data from statistics bureau also explain that types of land use have dramatically changed (Table 1). NDVI data showed that the greenest of the land cover changed from 2008 to 2012, with higher average of the NDVI changes in the eastern and southern parts (Figure 4).

Figure 4 clearly shows identified cluster areas after Sat-scan analysis. There were seven areas found in which four clusters have high Relative Risk (RR) in the period of 2009/1/1 to 2009/7/18 and one cluster in the period of 2013/1/3 to 2013/8/9. There were two areas identified as low risk clusters between 2008 and 2010. Four clusters with high RR associated with the outbreak in 2009, one cluster in 2013 showed that there was a change in the pattern of disease transmission. This change may be due to other demographic, environmental or behavioral changes over time in those areas. There was a significant correlation between IR and average NDVI in 2008 (R = −0.258; *p* = 0.001) and 2012 (R = 0.193; *p* = 0.017). Red area means The NDVI was increasing and green area means the NDVI was decreasing.

## 4. Discussion

Bandung was reported as hyper-endemic for dengue cases since continuous circulation of all serotypes of dengue viruses (DENV 1–4) where a large pool of susceptible host and complement vector are present. DENV 2 was identified as predominant among all serotypes in Bandung [5]. The population composition of Bandung shows expansive pyramid with dominant age group from 5–49 years old. This was consistent with DHF cases which are predominant in students and productive age group. Another study of dengue incidence in Indonesia showed that DHF incidence has increased substantially over the past 45 years with a pattern of intermittent hyper-endemic years. There was a steady decline in DHF incidence for children aged 5 to 14 years (the age group with the highest DHF incidence historically), while the incidence in those aged over 15 years steadily increased and surpassed the decreasing incidence in younger children since 1999 [3]. This pattern has also been observed in other endemic Southeast Asia countries, such as Thailand and Vietnam [9,10]. Shifts in modal age, rural spread, social and biologic determinants of race- and sex-related susceptibility also became point of interest. This result indicated that most of DHF cases may get the infection transmission during daytime when most of them were not at home. Since the mosquito is active and feeds during the day, this suggest that people might get infected when most of them are doing activity outside of their home (at school or at work).

Cases in this study were geo-coded based on their registered addresses in the hospitals. Incidence rate changes that is illustrated in Figure 3 is sequence to the fact that over the last decade in the southeast part of Bandung most of the land had been changed into housing, schools, office complex and recreation area. The extension of office complex, housing, residential areas in the eastern and southern parts of the city which is related to land use changes potentially increases mosquitos’ breeding sites. Some people who stay at home during the day will also be in higher risk if breeding sites are present in their home. Increasing residential areas also means increasing population density and breeding sites as well as increasing risk of contracting dengue. Moreover, increasing number of schools and office complex may also increase the risk for people who spend their daytime in school and work.

*Aedes aegypti* and *Aedes albopictus* are the main vectors for dengue infection. Theoretically, urban and indoor environment are in favor of *Aedes aegypti*, whereas rural, suburban and outdoor areas are in favor of *Aedes albopictus* [11,12,13]. This theory was slightly different from a study in Sri Lanka which found high prevalence of *Aedes aegypti* and *Aedes albopictus* in both indoor and outdoor area [14]. Consequently, this suggests that the prevention program should be re-oriented, so that vector control on the environment should not solely focus on the household and the community nearby, but also schools and workplaces should be prioritized—both inside and outside the buildings. The critical strategies for the prevention and control of DHF in Indonesia include surveillance system that cover both case and vector surveillances for planning and response, disease management and changing behavior and building partnership. Surveillance of *Aedes aegypti* is important to determine the distribution, vector density, major larval habitats, spatial and temporal risk factors related to dengue transmission and levels of insecticide susceptibility [15]. Primary health centers regularly conduct larval surveys every three months. However, the index from dengue surveillance program such as House Index, Container Index and Breteau Index were not uniformly measured in all areas, making it difficult to analyze overtime.

Aside from vectors, population density also needs our concern. Rapid urbanization, increased global trade and travel should be taken into account to facilitate the influence of higher DENV transmission between population of human and mosquito [16,17]. A study conducted in a municipality region in Brazil showed that female *Aedes aegypti* density was directly proportional to the number of residents in the houses. This may indicate a high probability of human-vector contact that increases the possibility of transmission [11]. Household density greater than three people per room is more likely to get dengue infection compared with those who live in less crowded accommodation [18]. Mosquitoes may spend their lifetime in or around the houses where they emerged as adults with flight distance of around 50–100 meters. On the other hand, we must consider that people act not only as a host but also one of the actors who ‘spread’ dengue virus within and among communities. Therefore, localized insecticide around a dengue patient’s home or community is not likely to prevent the spread of dengue infections.

A “disease cluster” is an unusually high concentration of disease in a region, which is unlikely to occur by chance. Spatial clustering of disease is almost inevitable since human populations generally live in spatial clusters rather than random distributions [19]. In the Asia-Pacific region, dengue cluster areas substantially increased from 1955–2004 with at least two countries joined the cluster areas every ten years and the tendency to spread in a southerly direction [20]. Clustering of dengue disease is also commonly found in medium or high populated area as seen in other countries like Cambodia, Thailand and Vietnam [19,21,22]. Clustering in medium-high populated area with spreading tendency to southern part of Bandung was consistent with these findings.

The use of remotely-sensed data and GIS such as NDVI should be implemented within the study of vector-borne diseases, especially in urban environments with spatial heterogeneity, complex movement of hosts and vectors and anthropogenic creation of vector habitats. Studies in Costa Rica and Colombia found that there were significant correlations between dengue incidence and urban structural variables. An increase in the occurrence of dengue cases was directly related to simultaneous deterioration of vegetation cover (vigor and density) in the area [23,24]. These results should be included as main consideration especially by the government and urban planners to improve dengue prevention and control.

### Limitations of the Study

Reported DHF cases before year 2008 (2003–2007) have been only in aggregate data (number of DHF cases/month). There is a chance of under-reporting in some areas that may be due to misclassified diseases or unreported cases from private clinics. Missing data were possibly accounted for due to incomplete registration or insufficient laboratory facilities. All hospitals should report new cases for hospitalized DHF patients every month to Bandung Health Office. However, only 18 out of 22 hospitals actively report the cases routinely. In addition to Primary health centers and hospitals, there are plenty of private clinics in Bandung that are organized by general or specialist physicians. However, not all of these clinics report their cases to the health office frequently. This implies that ecological fallacy may influence a part of the interpretation.

Vector control program in Indonesia have been introduced since 1992 through breeding site elimination programs by 3M movement (3M = *Menguras* (drain), *Mengubur* (bury), *Menutup* (cover)). Since year the 2000, the 3M Plus program has been socialized by additional activity to use larvacide, nurturing fishes in small ponds/water puddles and prevention of mosquito bites using mosquito repellent. This program was aimed to change the community behavior. To maintain this program, community empowerment should be encouraged. Bandung has a uniform surveillance method and vector control program, and each area follows the same regulation. The success of intervention programs may vary among areas, and this may also influence the changes in incidence rate overtime. However, this study does not analyze the differences of intervention program effect toward the changes in incidence rate.

## 5. Conclusions

Monitoring of cluster identification overtime would be valuable for targeting appropriate vector-control program in certain areas. However, regular evaluation in case reporting and improvement in dengue surveillance system by combining active and passive methods that cover all areas is needed to improve field data quality. NDVI as land use change indicator is potential for environmental risk factor in a rapid urban area. Land use development should consider the impact of dengue spreading and rapid urban development should be accompanied by an improved surveillance system.

## Figures and Tables

**Table 1:** Trend of land use change over time in Bandung.

Type of Land Use	Area (ha)			
	2008	2009	2010	2011
Wetlands	1727	1719	1474	1354
Garden/Wasteland	763	761	328	650
Farm	0	0	475	186
Housing, school, industry	7526	7538	6042	12,739
Office complex/Recreation	0	0	1854	1219
Pool/pond	72	70	70	35
Not used	0	0	0	185
Others	6641	6641	6458	363
